# “Preterm birth risk, me?” Women risk perception about premature delivery – a qualitative analysis

**DOI:** 10.1186/s12884-021-04068-x

**Published:** 2021-09-18

**Authors:** Thaís V Silva, Silvana F Bento, Leila Katz, Rodolfo C Pacagnella

**Affiliations:** 1 School of Medical Sciences, University of Campinas (UNICAMP), R. Alexander Fleming, 101. Cidade Universitária. Barão Geraldo, SP Campinas, Brazil; 2 University of Pernambuco, Recife, PE Brazil; 3 Women’s Hospital “Prof. Dr. José A Pinotti” – Center for Integral Attention to Women (CAISM), University of Campinas (UNICAMP), Campinas, SP Brazil; 4 Instituto de Medicina Integral Prof. Fernando Figueira (IMIP), Recife, PE Brazil

**Keywords:** Risk perception, Premature delivery, Pregnancy

## Abstract

**Background:**

Risk perception is based on collective indicators, but it is influenced by the individual’s self-perception of his health-disease process. This study aims to investigate the risk perception of pregnant women who were identified as high-risk for premature birth and to seek strategies for better management of such cases.

**Methods:**

This is a cross-sectional study where women who had completed their participation in P5 trial were contacted and invited to answer a structured questionnaire with open questions. Data were collected by telephone and analyzed using thematic analysis. The analysis categories were defined, and all the answers were reviewed, categorized, grouped, and a descriptive summary was prepared.

**Results:**

Two hundred eight Brazilian women have participated. Three categories were identified: (1) Risk perception mediated by health professionals; (2) Self-perception of risk through personal experiences and relationships; (3) Perception of treatment success. After receiving an explanation from a health professional about short cervix and premature birth, women understood the risk of premature delivery, recognizing the importance of early diagnosis to prevent premature birth. Unsuccessful previous experiences in prior pregnancies influenced women’s risk perception. Patients believed in the success of the treatment performed, placing their hopes on the treatment even without research guarantees about benefits.

**Conclusions:**

Pregnant women’s risk perception regarding prematurity is based partly on personal and family experiences but mainly on information given by health professionals. The risk perception about preterm birth may contribute to healthy pregnancy, guiding necessary interventions and preventing adverse outcomes. Prevention studies on prematurity should thus focus on neonatal outcomes.

**Supplementary Information:**

The online version contains supplementary material available at 10.1186/s12884-021-04068-x.

## Background

The development of risk perception is influenced by the individual’s self-perception of the health-disease process [1], which is characterized by the association with personal factors related to cognitive capacity, affective and biological aspects, and an ability to read and interact with the environment. Therefore, it is difficult to associate and scale the risk perception of patients and the influence of beliefs, values, meanings, attitudes, aspirations, motives and personal relationships in the outcomes [2, 3]. Thus, understanding and confronting risk will depend on one’s social and personal context and environmental pressures and demands [4].

The identification of risk factors and informing patients is part of the routine of health professionals. A patient’s risk perception of health guides decisions, openness to the proposed treatment and the results achieved. In critical situations, a patient’s self-perception of risk can further influence decisions. As an example, a woman can experience critical situations during pregnancy. Preterm birth, in turn, is one of the most important potential risks faced during pregnancy. Prematurity is the main cause of infant morbimortality, and it places a significant economic burden on the family and healthcare system due to the newborn’s need for higher levels of complexity in the provision of healthcare [5].

The high risk for preterm birth diagnoses during pregnancy can have negative effects on a woman’s quality of life, generating fear and anxiety about her future and her baby [6]. In view of the potential adverse events associated with premature births, this article aims to present the risk perception of pregnant women with a high clinical risk for prematurity. Our sample of female patients participated in a clinical trial that compared two interventions for preventing premature birth in order to identify possible strategies which could help with the management of patients exposed to risk situations.

## Methods

We developed a cross-sectional study based on the qualitative analysis of two open questions used to identify the risk perception of preterm birth with pregnant women who participated in a clinical trial entitled “A randomized controlled trial on the use of pessary plus progesterone to prevent preterm birth in women with short cervical length (P5 trial)” (Trial registration RBR-3t8prz) [7].

The P5 randomized controlled trial involves 936 pregnant women at a high risk of preterm labor and has been underway since July 2015 in 17 Brazilian centers. The main objective was to compare the efficacy of progesterone alone versus progesterone associated with cervical pessary for the prevention of preterm birth in pregnant women with a short cervix. All pregnant women from 18 to 22 weeks of gestational age attending antenatal care clinics at referral facilities were invited to participate in the study. The research team offered a transvaginal ultrasonography scan as a screening phase to identify the shortening of cervical length; according to the identification of a short cervix, which is an important risk factor for preterm birth, women were randomized to two treatment options. Following randomization, women were followed up until 10 weeks after the infant’s birth to evaluate neonatal outcomes. During the screening phase, all eligible patients received information about the risk of preterm birth by the research assistant and the medical doctor. All of those who agreed to participate signed an informed consent form. The Brazilian National Review Board (CONEP) approved the P5 trial under the number 1.055.555.

In order to identify possible difficulties to implementing a screening program for preventing prematurity, a descriptive cross-sectional study was conducted within the P5 clinical trial by applying a structured questionnaire with 36 queries, which included 8 open questions. These questions related to the women’s understanding of the doctor’s explanations, prematurity, risk perception, experiences of her participation in the study, doubts about whether it was appropriate for the woman to participate in the study and her perception regarding the success of the treatment provided.

**Fig. 1:**
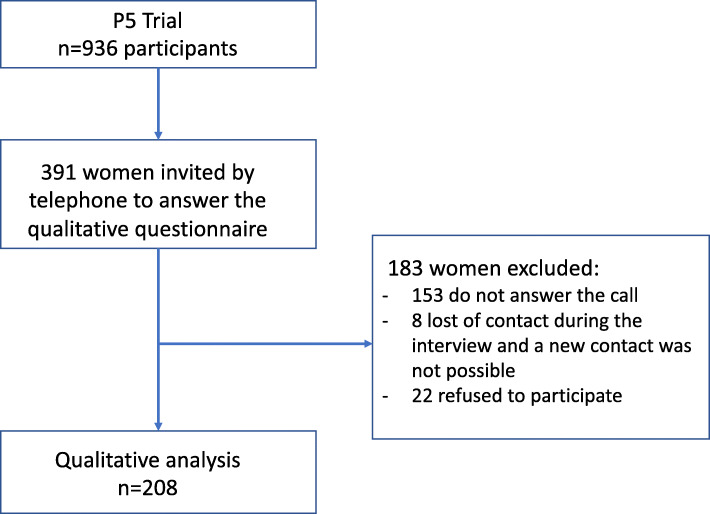
Patient enrolment flowchart

Patients who agreed to participate in the P5 trial were contacted after labor by telephone and invited to participate in the cross-sectional study. We tried contact with 391 women: 153 women could not be contacted by telephone, 22 refused to participate and 216 agreed to participate. For those, an interview was scheduled considering women’s convenience. During this second telephone contact, a total of 208 women completed all interview questionnaire. The interviews were conducted by telephone from January to July 2017 and an informed consent was given verbally and recorded after any doubts were clarified [8]. A complete patient enrolment is presented in Fig. 1. Each interview lasted for an average of 10 min and were recorded and registered in a digital version of the questionnaire, where the responses to the open questions were transcribed *ipsis litteris* into the database.

For the purposes of the present study, two open questions were analyzed: (1) what made you finally decide to undergo the proposed treatment; and (2) in your opinion, did the treatment work or not, and why? These two open questions answers were also analyzed in a previous publication to identify reasons given by pregnant women to participate in a clinical trial [9].

Data analysis followed Patton’s guidelines [10]. Units of analysis to achieve the study objectives were identified by two investigators after read all answers repeatedly. Using these units of analysis, the investigators defined specific categories and grouped all answers. This strategy allowed three categories of analysis to emerge for explaining how women identify and understand the risk of preterm birth: risk perception mediated by health professionals, self-perception of risk through personal experiences and relationships and perception of treatment success. This study was approved by the local and national Institutional Review Boards (CAAE 5592.3016.1.0000.5404).

## Results

### Sample characteristics

A total of 208 participants answered the full questionnaire. The mean age was 27 ± 6.7 years and 79.8 % women (166/208) lived with a partner. Most of the participants were non-white (60.6 %; 126/208) and 82.7 % had elementary or middle school level education. Considering obstetric history, 22.6 % had had a previous preterm birth. Regarding gestation outcomes, 64/208 participants had a preterm birth. There was one neonatal death for extreme preterm birth and one stillbirth. There were 12 readmissions to hospital and 75 % (9/12) were premature newborns.

### Risk perception mediated by health professionals

Overall, after a health professional provided a clear and detailed explanation about the diagnosis of a short cervix, the participants understood both the information given and the preterm birth risk factor. These participants were able to recognize the association between a short cervix and preterm birth and the importance of early diagnosis for preventing premature birth. Moreover, they were also able to understand that there are other risk factors for premature birth, such as twinning or other maternal morbidity, which together increase the risk of prematurity.

*“Oh based on what the doctor said, he said that my cervix is low and could not hold the weight of the baby” (patient 60)*.

*“…although I had a premature birth because of twinning and all that, so it was already expected” (patient 2)*.

Participants reported that the information given by health professionals involved in the P5 trial was impartial. The health professionals clarified that the P5 trial was a clinical research and that agreeing to participate would not guarantee that the participant would deliver at term. Participants reported that doctors said that *“….there is no conclusion if it* [treatment] *really works”* and that *“…maybe the treatment could not keep the pregnancy* [the baby in the womb]”.

The well-established relationship between the patient and the health professionals brought confidence in the treatment and safety to the patient on several occasions. Care, attention, answers to questions, trust and the excellence of the medical team were mentioned, including statements that the doctor was always very concerned about the patient, the feeling that the doctor was her *“lifeboat”*, and that having access to the doctor by phone at any time to solve the patients’ doubts, was a good experience. One participant even considered that all monitoring and perinatal care from P5 trial health professionals was as responsible for the baby’s health as the treatment itself.

*“…the doctor’s quality, treatment, care, the way he explained to me about the treatment, his follow-up for months and after the treatment. For me, I think he was the main factor responsible because of his treatment, care, concern and guidance. Do you know what I mean? For me, in addition to progesterone, it made me successful” (patient 170)*.

Most participants reported feeling gratitude for the health professionals who accompanied them during pregnancy and the opportunity to participate in the P5 study.

*“I have to say that it was an amazing experience [the treatment], I really appreciate it. The doctors are all attentive, they are wonderful; the medical team is to be congratulated, the study team as well” (patient 160)*.

Many participants also thanked the study for the opportunity to receive a short cervix diagnosis during their first pregnancy, without having to go through the experience of having a premature child before being diagnosed.

*“I would like to thank [the study] for this opportunity…it helps me a lot because I know that I have a short cervix; it was something new to me and I received help…thank God I got to the end and I didn’t have a preterm baby” (patient 116)*.

### Self-perception of risk from personal experiences and relationships

The participants who had an unsuccessful experience in previous pregnancies, such as miscarriage, stillbirth, neonatal death, premature birth or having the baby admitted to a Neonatal Intensive Care Unit (NICU), associated their history with the informed risk in the current pregnancy, influencing their self-perception of risk. This created concern and anguish, because these participants did not want to go through that suffering again. One participant reported that *“my other babies* [previous gestations], *it was so hard following them in the NICU”*, so her wish was to “*leave* [the hospital] *with the baby in her arms”.*

Family experiences have also influenced the participants’ decision to accept treatment. Having someone close to them who had suffered an adverse outcome during their pregnancy helped women to recognize preterm birth adverse events, and stimulated a feeling that something similar could happen to their baby.

*“…I had a case of preterm birth in my family and the baby died because he was born at 5–6 months, so I didn’t think twice. I agreed right away (to participate in the P5 trial); I said that if it will keep my baby inside my womb, I will accept it until the end” (patient 78)*.

### Perception of treatment success

Although P5 study has not yet demonstrated the higher efficacy of one treatment in relation to the other, and considering that the literature is controversial regarding the effectiveness of treatments used to reduce premature birth incidence, overall, patients considered that the offered treatment to avoid premature childbirth had a positive result, and that it worked properly. Even those who experienced a premature birth considered that the treatment worked, because the birth would have happened earlier than it did without this treatment. The feeling of successful treatment is clear in phrases like *“it worked because if I hadn’t done it, I could have lost my son”* or “*I felt like it was my salvation*”. Patients seem to project their hopes on the treatment, waiting for any benefit that it may bring to their pregnancy.

For some participants, even without reaching a term pregnancy, the treatment was able to prolong gestation, which would have brought many benefits to their babies’ survival. The participants understand that neonatal outcomes influenced for prematurity depend on gestational age at the time of delivery and that the closer to a term gestation, the lower the risks would be.

*“I had a previous pregnancy and hadn’t done this treatment, so I didn’t even get to 30 weeks. Thus, I lost my baby. In this gestation, I maintained my pregnancy to 33 weeks with the treatment, and my baby was born; now he is fine, he is very strong” (patient 14)*.

Most participants reported that the treatment proposed was able to “*keep the baby until the appropriate time*”, while others mentioned that having their baby healthy at the time of the interview is already the answer to the question, because “*it worked because my baby is in my arms right now*”. Another reported factor was a decrease in some patient symptoms such as pain, bleeding and an increased feeling of security and tranquility. These feelings were cited not only as a consequence of performed treatment, but also as being responsible for the treatment success.

*“It worked [the treatment] well. It worked because I have my son with me, well and healthy, without any risks. Do you know what I mean? It worked because I kept my pregnancy safe until the end” (patient 47)*.

*“After I started [the treatment], the bleeding stopped, and the pain also stopped, so it worked for me. Also, I left the risk area and my pregnancy became stable” (patient 64).*

Participants stated that they had undergone successful treatment, mainly by comparing the current pregnancy with their previous experiences. They also reported their personal experience of having a previous baby on NICU and all of the suffering linked to this moment of uncertainty, regarding whether the premature newborn would survive. Many participants reported that they could carry the pregnancy to term with the managed treatment. Few patients believed that the performed treatment did not work, and all of them reported stillbirth or neonatal death as the final result of the pregnancy. Half of these neonatal outcomes were linked to extreme prematurity. Therefore, the perception of failure is strongly connected to the baby’s death.

*“No, it didn’t [the treatment didn’t work], because I have not been successful. If I hadn’t done this treatment, the same thing would have happened” (patient 29)*.

Participating in the research was also emphasized, due to the possibility of receiving adequate treatment. Some women said that they would redo the treatment and others reported that they would recommend the treatment to relatives or friends.

## Discussion

Overall, pregnant women participating in this study recognized the preterm birth risk to which they were exposed from personal experience and information provided by the health team. The image of good health and female fullness associated with the pregnancy process is broken after the diagnosis of a risk condition, breaking projections and expectations launched an expected positive birth outcome. Recognizing illness is different from recognizing the presence of a disease, and this will influence how well the patient will follow the proposed treatment.

It is clear that, in this study, knowledge of the risk of prematurity was also influenced by their own previous experience and/or relationships [11]. In particular, when a patient has a history of premature birth, it is natural that she asks the doctor about prematurity and the possibility of going through this negative event again, since she brings with her a risk perception of prematurity formed through her previous experience.

Having access to medical criteria defined by doctors as a disease condition and the way in which they were informed interfere with the process of recognizing this risk condition; it brings the patient’s perception of illness closer to the illness process [12]. In addition to the easy understanding of information, participants in the present study also praised the way in which it was transmitted. The study results reinforce the need to invest in health education as a way to offer autonomy, empowerment and health literacy [13, 14]. Within the collective, the external social influence on the individual’s risk perception can be seen in the valuation of certain diseases. Considering pregnancy, we can exemplify the need for health education with the current Brazilian population perceptions of risk on prematurity and congenital Zika syndrome. Although prematurity is the main cause of infant morbidity and mortality worldwide, one of the greatest fears of families in our country is currently linked to the possibility of Zika virus infection during pregnancy, or more directly, linked to microcephaly, despite the lower incidence [15].

Developing a risk perception of prematurity, patients understood the information provided about the diagnosis; they individually recognized the disease and chose to participate in the research, accepting the proposed treatment based on their self-perceptions of risk. In this study, it is noteworthy that pregnant women develop an appropriate knowledge of prematurity. They were able to understand that the closer they were to term gestation, the lower the associated risks would be.

The participants who, even with the treatment, still had a premature delivery, but gave birth to a healthy baby and who had a premature birth at a lower gestational age in the past, attributed this higher gestational age and better outcomes at birth to the offered treatment, even though they had not reached full term gestation. Expecting positive neonatal outcomes, women may believe that any performed intervention is beneficial and capable of reducing the risk.

The follow-up throughout this study provided a good doctor-patient relationship, strengthening this bond and reducing, within the patient condition of vulnerability, the fear and anxiety created by the pregnant woman’s knowledge of her high risk of preterm birth. Many participants who initially felt fearful about the future of their pregnancy, considered the medical follow-up and treatment offered by the study as a divider; this changed their position as high-risk pregnant women into a group where this risk could be “controlled” or even “removed” [16]. On the other hand, participation in a study or the agreement to use some treatment, even without clinical evidence, can create a false sense of security.

Although participants were informed that this treatment was not proven to be effective and that participating in the study would not guarantee positive results regarding their health or that of their fetus, many still believed that they would no longer be at risk from the moment they started to receive the treatment. During pregnancy, women can experience a critical and vulnerable situation and can have their actions driven by anxiety and fear. In this way, women place hope and faith into the health professionals and in the treatment offered by them, although there were no guarantees of results.

This fragility associated with the lack of health professional proper conduct can increase the number of interventions and over-medicalizations in pregnancy, which are often unnecessary and even harmful [17, 18]. Examples of damage to the patient’s health are situations in which their decisions were guided by a false perception of risk, based on and induced by knowledge from health professional information that was not the result of scientific evidence, such as unnecessary cesarean sections which are common in Brazil [19]. Therefore, it is important that health professionals are clear and individualize each patient’s risk, avoiding unnecessary interventions without scientific evidence looking for unfounded protection to their patient health.

The search for health protection also emerges as the patient’s focus. The child’s good health at the time of interview was cited as proof of the success. However, participants who experienced stillbirths or neonatal deaths considered the treatment ineffective, even though their gestational age at birth was higher than expected. These results describe the real primary outcome expected by patients exposed to the risk of prematurity: a healthy baby. For participants who still had premature babies, regardless of gestational age or length in the NICU, but which had a positive neonatal outcome, the association with satisfaction and success is clear.

This identifies an important fact: studies for preventing preterm birth must consider neonatal outcomes as the primary outcome, rather than just focusing on higher gestational ages at birth. It will increase the quality and importance of these studies [20, 21]. In practice, increasing gestational age as a primary outcome for prematurity studies is an intermediate outcome that does not achieve the study participants’ goals. Thus, we believe that studies to prevent premature delivery should bring neonatal outcomes as primary outcomes, achieving the desire of pregnant women exposed to prematurity.

Health professionals are the most important element in the risk perception of pregnant women and must to consider that the concept of risk involves failures. In this sense, communication about risk must be careful and should not cause alarm and suffering. We must consider how much it is worth patterning all behaviors avoiding a future risk that we cannot be sure will occur. It is important to recognize that there is a need to establish a more fluid and horizontal relationship between patients and health professionals. Therefore, patients will have more autonomy in this relationship, incorporating their risk perception and opinions to their decisions, respecting patient individuality and leading to a more satisfying experience [22].

Regarding the limitations of this study, it is a qualitative analysis with an exploratory characteristic; therefore, the results should not be extrapolated beyond its limits. Although we showed that participants reported that they understood the information about preterm birth risk, this information was not checked in a systematic way. Also, the fact that participants were interviewed weeks or months after delivery, which can limit their memories about occurred facts and, therefore, their comments can also be considered a limitation. Another important issue is the fact that most women who refused to participate had a negative outcome (miscarriage, stillbirth or neonatal death), which reduces the number of women interviewed who had an unfavorable outcome.

There are still few studies about pregnant risk perception in the face of a critical situation such as prematurity; therefore, it is not known whether they understand or have experienced this risk yet. Recognizing an existing risk in pregnancy can further increase the vulnerability of pregnant women, creating a stressful situation regarding uncertainty about the future of the pregnancy, as well as helping to find the best way to deal with the risk, and providing the possibility of receiving adequate prenatal care, sharing decisions and responsibilities and increasing the acceptability of the proposed treatments.

## Conclusions

Pregnant risk perception about prematurity is based on personal experiences and mainly by clarifications made by health professionals. This individual assumes the role of knowledge about risks involved in pregnancy. However, it is important to emphasize that the concept of risk is not limited to numerical calculations. Risk is a social construct and associated with possibly unwanted events; its severity or risk cannot be represented by only collective, objective and numerical data.

Extrapolating risk use can generate distortions, misunderstandings and even suffering for women, creating a false sense of prevention and the control of threatening situations in both our lives and health, and providing scope in this process for medicalization and unjustified interventions, which must to be avoided when considering the patient’s individuality.

Risk perception, when it is well supported and reinforced by health literacy, can help pregnancies to progress properly, where women can share decisions with the health team and implement any necessary actions. Moreover, we recommend that new clinical trials to prevent prematurity must focus on neonatal outcomes in order to achieve families’ expectations.

## Supplementary Information

**Additional file 1.**

## Data Availability

The datasets analyzed during the current study are available from the corresponding author upon reasonable request.

## Abbreviations

P5 trial: Pessary plus progesterone to prevent preterm birth in women with short cervical length
CONEP: The Brazilian National Review Board
NICU: Neonatal Intensive Care Unit
